# International trends in shoulder replacement: a meta-analysis from 11 public joint registers

**DOI:** 10.2340/17453674.2024.40948

**Published:** 2024-06-18

**Authors:** Neal RUPANI, Christophe COMBESCURE, Alan SILMAN, Anne LÜBBEKE, Jonathan REES

**Affiliations:** 1 Nuffield Department of Orthopaedics, Rheumatology and Musculoskeletal Sciences, University of Oxford, Oxford, UK; 2 Division of Orthopaedic Surgery and Traumatology, Geneva University Hospitals and University of Geneva, Geneva, Switzerland; 3 Division of Clinical Epidemiology, University of Geneva and Geneva University Hospitals, Geneva, Switzerland

## Abstract

**Background and purpose:**

International variation exists in the types of shoulder replacement used for treatment of specific diseases. Implant choice continues to evolve without high-quality evidence. Our aim was to evaluate trends in incidence rates of shoulder replacement and assess any recent changes in practice between countries by using registry data.

**Methods:**

Patient characteristics, indication and year of surgery, type of replacement, and collection methods of patient-reported outcomes (PROMs) was extracted from 11 public joint registries. Meta-analyses examined use of reverse total shoulder replacement (RTSR) for osteoarthritis, cuff tear arthropathy, and acute fracture; use of anatomical total shoulder replacement (TSR) for osteoarthritis; and use of humeral hemiarthroplasty for fracture.

**Results:**

The annual growth rate of shoulder replacements performed is 6–15% (2011–2019). The use of RTSR has almost doubled (93%). RTSR is now universally performed for cuff tear arthropathy (97.3%, 95% confidence interval [CI] 96.0–98.1). Its use for avascular necrosis, trauma, and inflammatory arthropathy is increasing. The use of RTSR was similar (43.1%, CI 30.0–57.2) versus TSR (44.7%, CI 31.1–59.1) for osteoarthritis. The types of PROMs used, collection time points, and response rates lack standardization. COVID-19 had a varying inter-registry impact on incidence rates.

**Conclusion:**

The incidence of shoulder replacements has grown. Use of RTSR has increased for all disease indications despite limited high-quality evidence driving this change in indications outside of cuff arthropathy. Consequently, less variation is observed in international practice. Existing differences now relate to use of newer implant types and methodology of PROMs collection, which prevents international comparison and outcome analysis.

Different types of shoulder replacement aim to provide a surgical solution to different disease indications in order to improve pain and function. The main types of shoulder replacement include anatomic total shoulder replacement (TSR), reverse total shoulder replacement (RTSR), and humeral hemiarthroplasty (HH). These can be subdivided into resurfacing, stemmed, and stemless replacements. The most common indications for shoulder replacement are osteoarthritis (OA), cuff tear arthropathy, inflammatory arthropathy, acute trauma, trauma sequalae, and avascular necrosis (AVN). More recently RTSR has been used for irreparable rotator cuff tears with functional loss but without arthritis.

International data has previously demonstrated wide variation between countries in the type and use of shoulder replacements [1]. High-quality evidence to guide the choice of what type of shoulder replacements to use remains sparse [2-4]. Despite this, there have been technology advances and growing confidence in some shoulder replacement types, potentially leading to change in surgical practice without high-quality evidence.

The primary aims of this study were (i) to assess the incidence of shoulder replacement surgery in the different countries, (ii) to evaluate whether there have been any temporal changes in patient characteristics, indication for surgery, and type of replacements, and (iii) to assess for any changes in international variation in the type of replacements used for different indications.

Secondary aims were to assess inter-registry variation in PROMs collection and to document the impact of COVID-19 on the provision of shoulder replacements between countries.

## Methods

All regional or national registries within the International Society of Arthroplasty Registries with publicly available shoulder replacement reports were identified [5]. Annual implant-specific data up to the year ending December 31, 2019 was required for inclusion in order to mitigate any COVID-19 risk bias. National registry data was included from Australia, Norway, UK, New Zealand (NZ), Denmark, Sweden, and the Netherlands [6-12]. Regional registry data included were Emilia-Romagna (Italy), and multi-regional registries by the American Academy of Orthopaedic Surgeons (AAOS, USA) and Germany [13-15]. 3 publications based on national registry data from Italy, Denmark, and Germany were used only to assess national incidence trends [16-18]. Additional reports published that included procedures performed from 2020 to 2022 were only used to assess the impact of COVID-19 on the annual incidence of shoulder replacement and included registry data from the UK, Australia, NZ, Denmark, and the Netherlands. The Kaiser Permanente registry (California, USA), and Finland were excluded as they had not published reports in 2019 [1,19]. The Italian Arthroplasty Registry was omitted due to low completeness of 46% [20].

### Data

Demographic data was extracted. Race and ethnicity data was not available. Completeness, which is the percentage of procedures performed picked up in the registry against those truly performed as per hospital episode statistics, was listed for national registries to document adequate national representation. Based on primary shoulder replacements, the 4 variables collected were year of surgery, type of procedure, disease indication, and collection of patient reported outcomes. The data was used to assess the following.

***Incidence:*** Annual national population data was gathered from each country’s equivalent version of an Office of National Statistics [21-27]. The annual incidence was calculated as the number of shoulder replacements performed per 10^5^ population. Annual incidence data from after January 1, 2020 was used to assess the response to COVID-19.

***Distribution of disease indications and types of replacement:*** Distribution was calculated as a percentage of all primary replacements performed in that registry. Disease indications were OA, rotator cuff tear arthropathy, inflammatory arthritis, AVN, acute trauma, and trauma sequelae. Indications such as instability, tumor, and infection were excluded. The Netherlands had separate indications for rotator cuff tear and rotator cuff arthropathy, which were combined. Types of shoulder replacement were split into RTSR, TSR, HH, and Resurfacing. Data was extracted and analyzed where available.

***Trends over time:*** For assessment of temporal variation, the number of each type of replacement performed at 4 time periods (2000, 2006, 2014, 2019) was extracted, allowing direct comparison with a previous variation paper [1]. If data for the specific year was not available, data from a year either side was allowed, except for 2019 where data from 2020 was not permitted.

***Patient-related outcome measures:*** Data was collected on the specific time points of any PROMs collection, the specific PROMs used, the completeness of data and how completeness was calculated. PROMs results were not collected. No quantitative analysis was performed.

### Statistics

Incidence rate ratio was calculated between the country with the highest and lowest incidences. Average compound growth in incidence was calculated based on incidence data from 2010 and 2019, although where 2010 data was not present, the closest year was used (UK—2012, Germany—2009, Netherlands—2014). Confidence intervals (CI) were set at 95%.

Meta-analyses were conducted to assess (i) inter-registry variation in indication-specific implant choice and (ii) combined proportions of TSR performed for OA, RSTR performed for cuff-tear arthropathy, OA and acute fracture separately, and HH performed for fracture. A generalized linear model with random effects was used, assessed with the restricted maximum likelihood approach [28]. Between-registry variability was assessed by the Tau2 and I2 statistics [29,30]. The magnitude of variability was demonstrated with the 95% prediction interval [31]. Leave-one-out sensitivity analyses were conducted to identify influential registries. Meta-analyses were conducted with meta version 6.1-0 for R version 4.0.2 (R Foundation for Statistical Computing, Vienna, Austria).

### Ethics, funding, and disclosures

No specific ethical approval was required for this study. No funding was sought for this study. There are no conflicts of interest from any authors. Complete disclosure of interest forms according to ICMJE are available on the article page, doi: 10.2340/17453674.2024.40948

## Results

### Annual incidence of shoulder replacements performed in national registries

**Figure 1:**
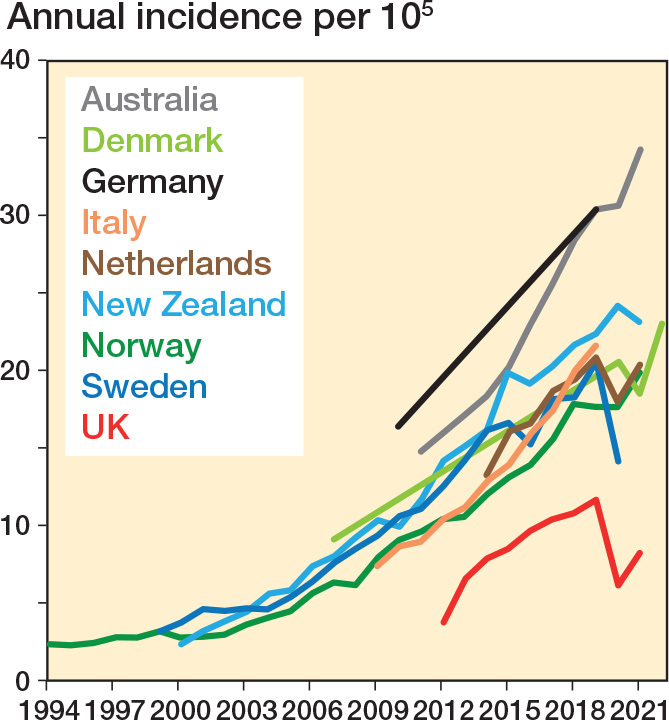
Annual incidence of shoulder replacement.

The annual incidence of shoulder replacement increased in all countries up to 2019 (Figure 1) ranging from 11.8 (UK) to 30.4 (Germany) procedures per 10^5^, difference 18.7 (CI 18.3–19.2, P < 0.001) (Table 1). The incidence rate ratio between these countries was 2.6 (CI 2.5–2.7, P < 0.001) The compounded annual growth rate from 2010 to 2019 ranged from 6% in Germany to 15% in the UK. The UK had the lowest incidence, but the highest rate of growth.

**Table 1:** Incidence (per 10^5^ inhabitants) of shoulder replacement performed in 2019 from national registry data

Country	Population (millions)	Prostheses	Incidence
Australia	25.5	7,735	30.3
Denmark	5.8	1,195	20.6
Germany	83.2	25,294	30.4
Italy			21.7
Netherlands	17.4	3,619	20.8
New Zealand	4.9	1,104	22.4
Norway	5.3	944	17.7
Sweden	10.4	2,140	20.6
UK	66.8	7,804	11.7

6 countries had data on shoulder replacement provision during the COVID-19 pandemic. The UK (47%) and Sweden (31%) had the biggest reduction in provision during the first pandemic year. The incidence in the first pandemic year was higher than the preceding year in Australia and NZ, growing by 1% and 8% respectively. Only NZ matched its growth rate in previous years.

### Comparison of inter-registry demographics

Demographic data from the 11 included registries demonstrated high coverage between 89% in UK and 100% in Australia (Table 2), which is defined as the percentage of procedures identified in the registry compared with the number of procedures actually performed. There was no change in mean age over 7 years (Table 3). There was a continued higher ratio of procedures in women, ranging from 1.5:1 in Italy to 6:1 in Sweden, although since 2014 the proportion of surgery in women in all countries dropped by between 1% and 4%.

**Table 2:** Demographic data of national and regional registries

Country	Years included	Total no. included			Procedures in women (%)	Mean age			Coverage (%)
		Primary	Revision	All		Women	Men	All	
National registries									
Australia	2004–2021	69,243	7,104	76,347	61	73.4	70.2	72.2	100 ^e^
Denmark	2004–2022	17,999	1,339	19,338	68	72.9	66	70.2	92.3
Finland	2004–2015	7,504		7,504	65	67	67	67	97
Italy ^a^	2009–2019	73,046	2,129	75,175	60	72.8	67.5	71.5	
Netherlands	2014–2021	22,536	2,412	24,948	74			71.5	97
New Zealand	2000–2021	13,072	744	13,816	61	72.7	68.5	71	>95
Norway	1994–2021	11,286	1,085	12,383	69			70.2	90.8
Sweden ^b^	1999–2020	24,039	2,049	26,088		73	71.5	72	>94
UK	2012–2021	56,312	1,902	58,214	77			73	89
Regional registries									
AAOS (USA)	2015– 2021	10,302		10,302		71	68.5	69.8	NA ^a^
Germany	2006–2019	9,260	266	9,526	66			70.8	NA ^c^
Emilia-Romagna, RIPO (Italy)	2008–2019	8,185	587	8,772	71	64.9–73.6	60.4–70.1		96.9 ^d^

a Regional voluntary data, so coverage data not available/deducible.

b Mean ages approximated from linear graph available in registry.

c Regional registry, including 70 institutes; coverage data not available/deducible.

d Regional registry, coverage quoted directly in paper.

e Full country collection since 2008.

**Table 3:** Comparison of age and sex distribution over time

Country Year	n	Mean age	Women (%)
Australia			
2014	2,4163	71	62
2021	69,243	70	61
Denmark			
2014	9,061	69	70
2022	17,999	70	68
Emilia-Romagna			
2013	2,881	71	73
2019	8,185	–	71
New Zealand			
2014	6,331	71	64
2021	13,072	71	61
Norway			
2014	5,621	70	73
2021	11,286	70	69
Sweden			
2013	11,414	69	65
2020	24,039	72	–
UK			
2014	11,399	72	72
2021	56,312	73	69

### Indications for surgery and types of shoulder replacement performed in each registry

**Figure 2:**
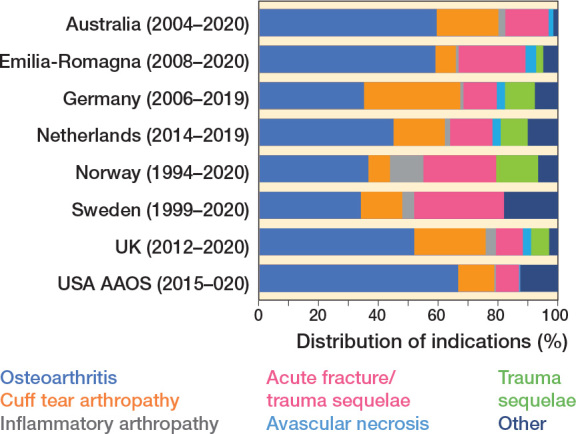
Distribution of indications for shoulder replacement.

The most frequent indications for surgery were OA (58%), cuff arthropathy (18%), and acute fracture (17%) (Figure 2 and Table 4, see Appendix). OA was the most common indication in all countries. Only in Norway, Denmark, and Sweden, the 3 longest running registries, was replacement more common for acute fracture than cuff arthropathy. The number of shoulder replacements performed for cuff arthropathy increased in all countries (Table 5, see Appendix).

**Table 4:** Distribution of indications for shoulder replacements performed through the lifetime of the registries. Values are count and percentages

Country	Total, n	Osteo-artthritis	Inflammatory arthropathy	Cuff tear arthropathy	Acute fracture/trauma sequelae	Avascular necrosis	Trauma sequelae	Other
Australia 2004–2020	53,790	59	2.2	21	14	1.6		1.5
Denmark (2004–2020)	15,260	36	3.3	15	39	2.8		4.9
Germany (2006–2019)	8,884	35	1.1	32	11	2.8	9.9	7.7
Emilia-Romagna (2008–2020)	8,581	59	0.9	6.9	22	3.6	2.3	4.9
Netherlands (2019)	3,245	45	1.9	17	14	2.8	9.0	10
Norway (1994–2020)	10,085	36	11	7.4	24	0.0	14	6.7
Sweden (1999– 2020)	22,189	34	4.0	14	30			18
UK (2012–2020)	48,401	52	3.5	24	9.0	2.8	6.0	2.9
USA AAOS (2015–2020)	5,760	66	0.5	12	7.6	0.5		13

**Table 5:** Proportion of shoulder replacements performed for cuff arthropathy over time

Country Year	Primary interventions for cuff arthropathy, n	Proportion of all indications (%)
Australia		
2014	3,551	15
2021	11,226	21
Emilia-Romagna		
2013	50	1.7
2019	590	6.9
Norway		
2014	101	1.8
2021	744	7.4
Sweden		
2013	839	7.4
2020	3,106	14
UK		
2014	2,343	21
2021	11,608	24

RTSR represented over 50% of all shoulder replacements in all registries except for Norway and Sweden (Table 6, see Appendix). Sweden and Norway had a higher proportion of HH performed compared with all other registries. The combined proportion of all resurfacing was low throughout all registries, at between 1% (AAOS, USA) and 7% (UK).

**Table 6:** Distribution of types of shoulder replacements performed through the lifetime of the registries. Values are count and percentages

Country	Total, n	Anatomic total shoulder replacement	Resurfacing	Humeral hemi-arthroplasty	Reverse total shoulder replacement
Australia (2004–2020)	53,790	30	3.5	9.7	56
Emilia-Romagna (2008–2020)	8,581	7.3	1.5	14	78
Germany (2006–2019)	8,884	28	2.0	8.7	62
Netherlands (2019)	3,245	19		7.3	74
Norway (1994–2020)	10,085	19	4.6	34	42
Sweden (1999–2020)	221,89	32	3.1	36	30
UK (2012–2020)	48,401	30	7.1	11	52
USA AAOS (2015–2020)	5,760	36	0.6	0.1	63

### Distribution of types of shoulder replacement performed for disease-specific indications

**Figure 3:**
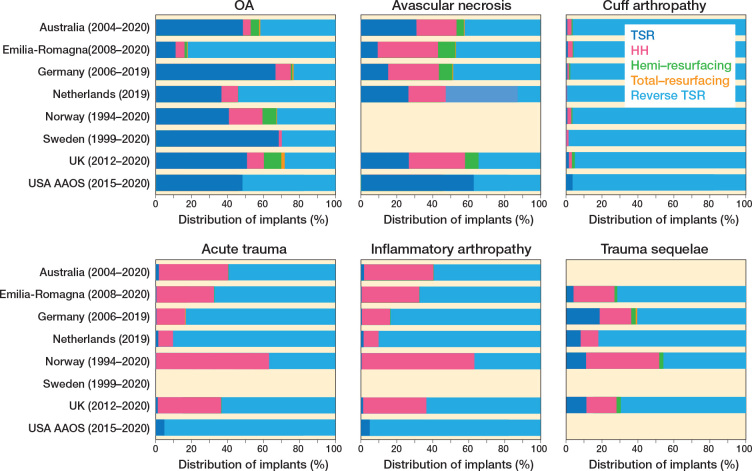
Distribution of types of shoulder replacement procedures performed across registries for 6 specific indications.

**Figure 4:**
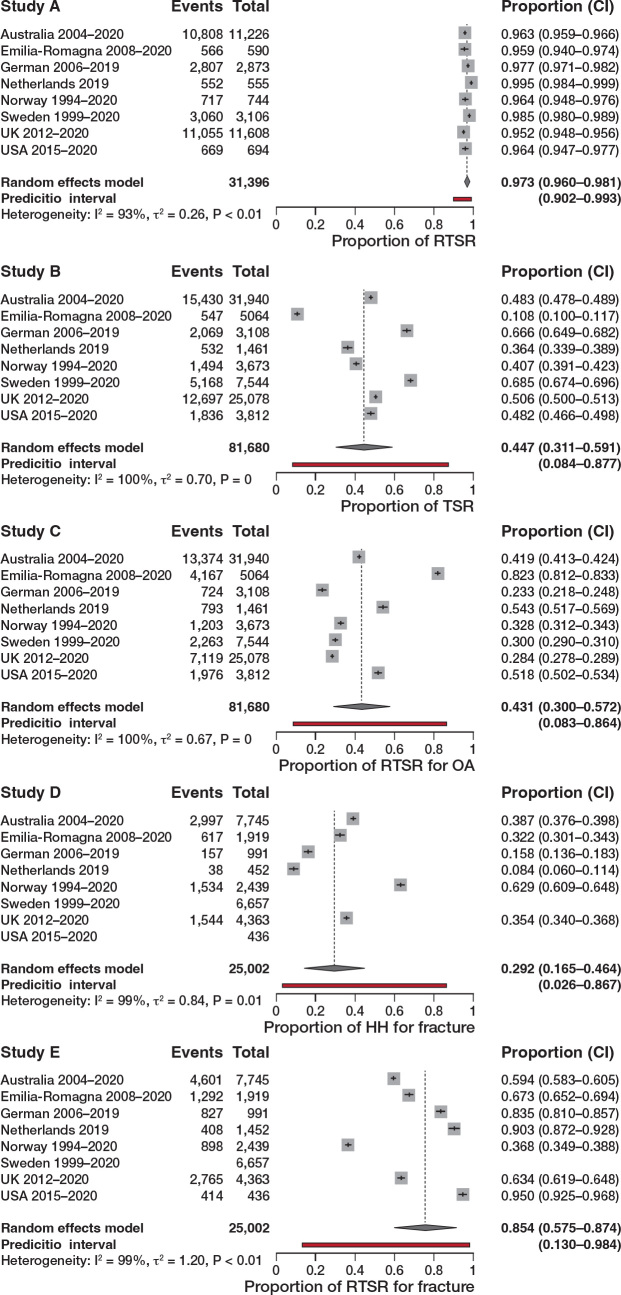
5 different variation meta-analyses displayed as a forest plot for different procedure types for specific indicators to assess variation between registries.

With regard to change in indication-specific implant choice (Figure 3 and 4), RSTR is now used universally for cuff tear arthropathy (97% meta-analysis pooled proportion, 90–99% new registry prediction), suggesting no variation between registries for this disease indication. This was unchanged with leave-out analysis.

An increased use of RTSR for primary glenohumeral osteoarthritis over 5 years was also observed. The pooled proportion of use of RTSR for OA was 43% compared with 45% for TSR, with both meta-analyses demonstrating wide prediction intervals despite leave-out analysis, demonstrating variability and equipoise in choosing RTSR or TSR for OA.

There was a significant difference in use of RTSR (75%) and HH (29%) for acute fracture with wide prediction intervals, highlighting preference for RTSR but ongoing national variability in choosing implant type.

### Change in types of shoulder replacement performed over time

**Figure 5:**
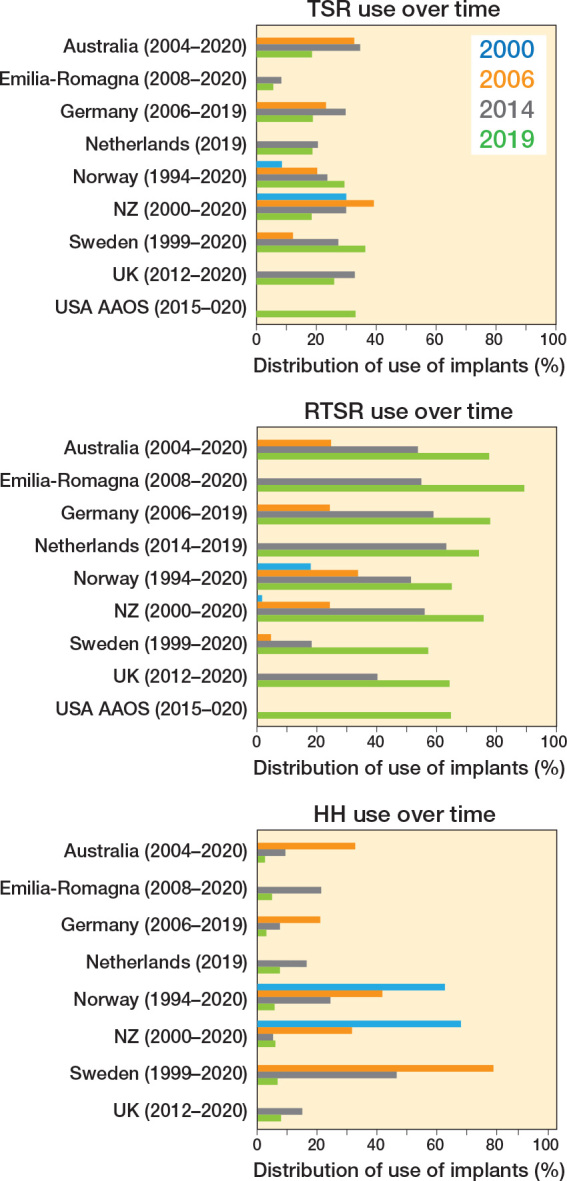
Proportion of types of procedure performed over time.

The distribution of implant types over time shows that RTSR use has grown universally, and is now the dominant shoulder replacement performed (57–89%) (Figure 5). The annual combined number of RSTRs performed (6 registries) almost doubled from 7,963 (2014) to 15,398 (2019). The proportion of RTSR for inflammatory arthritis, AVN, acute fracture, and trauma sequelae has also increased. Norway had a lower cumulative proportion of RTSR performed, but the proportion of RTSR performed in 2019 was within range of the other registries.

In contrast, the proportion of HH declined in all registries to a low level by 2019 (3–8%). The proportion of TSR varied, with Norway and Sweden showing a rise in its proportion, but with all other registries showing a fall over the last 5 years.

### Use of new stemless humeral components

**Figure 6:**
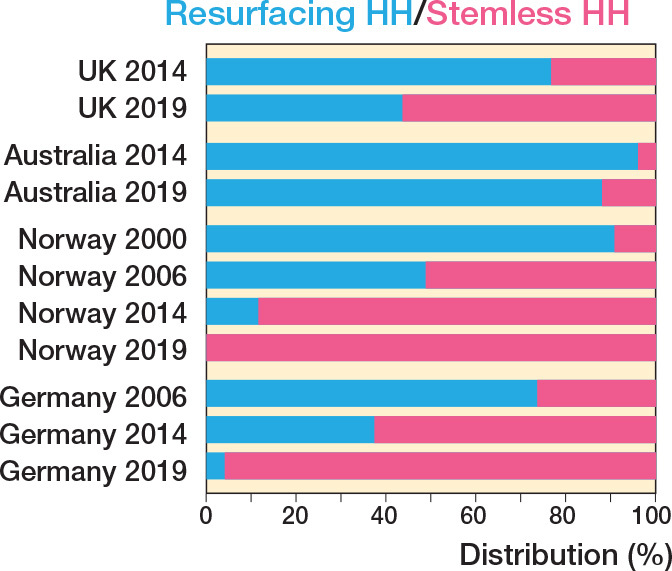
Proportion of resurfacing humeral hemiarthroplasty performed against stemless hemiarthroplasty performed over 4 time intervals.

**Figure 7:**
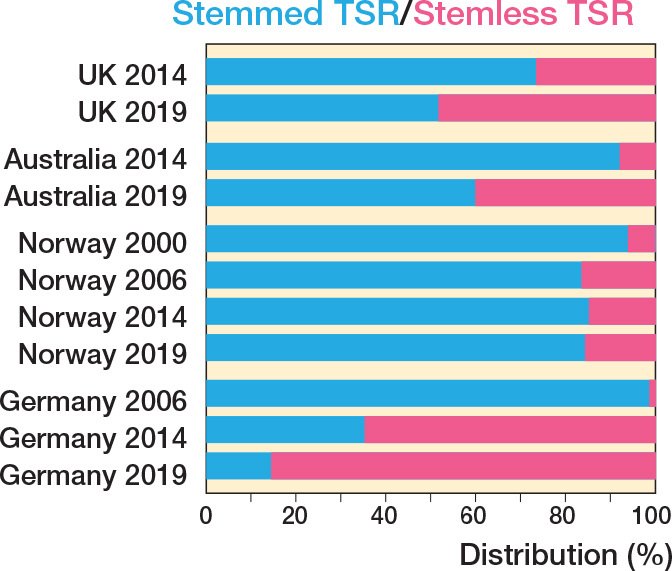
Proportion of stemmed versus stemless total anatomical shoulder replacement performed over 4 time intervals.

The UK, Germany, Norway, and Australia publish stemless humeral implant data for TSR and HH, with the former 2 also publishing stemless data for RTSR. More stemless HHs are now performed than resurfacing HHs, which have declined (Figure 6). The uptake of stemless TSR is variable (Figure 7), with only Germany having a strong preference for stemless humeral implants (86%).

### Variation in PROMs collection

Not all registries collect PROMs. Among those that do, there is variability in PROMs’ short- and long-term completeness rates, calculations of completeness and reporting (instrument used, timing) (Table 7). The most frequently used condition-specific PROM was the Oxford Shoulder Score (OSS), used in 4 countries (Australia, New Zealand, Netherlands, UK), followed by the generic PROM EQ5D in 3 countries. Early postoperative PROMs were collected either at 6 months (4 registries) or at 1 year (4 registries). The most frequently reported time point at later follow-up was 5 years.

**Table 7:** Qualitative assessment of patient-reported outcome scores collected and their completeness

Country	PROMs	Time collected	Preoperative completeness	Postoperative completeness
Australia (2004–2020) ^a^	EQ5D, OSS, EQ-VAS, Satisfaction/improvement questions	6 months	51–100% ^d^	6 months (34–89%) ^d^
Denmark (2004–2019) ^c^	WOOS	1 year		1 year (68%)
Germany (2006–2020) ^a^	Constant score,	Preoperative,	51%	6 months (29%),
	DASH score, ROM	3 months, 6 months, 1, 2, 3, 4, 5 years		5 years (11.5%)
Emilia-Romagna (2008–2020)	No PROMS			
Netherlands (2014–2020) ^c^	NRS, EQ5D, OSS, Anchor questions	Preoperative, 3 months, 1 year	40.5%	
New Zealand (2004–) ^b^	OSS	6 months,	–	6 months (72.2%),
		5, 10, 15 years		5 years (24.5%)
Norway (1994–2020)	No PROMS ^e^			
Sweden (1999– 2020) ^a^	WOOS, EQ5D	Preoperative, 1, 5, 10 years	48%	3 years (82%), 5 years (72%)
UK (2012–2020) ^a^	OSS	Preoperative, 6 months, 3, 5 years	23%	6 months (32%), 3 years (9%), 5 years (11%)
USA AAOS (2015–2020) b	PROMIS-10 Global, VR 12, ASES, SANE	unknown		8.2%

a Completeness calculated as percentages of the number of patients who returned their preoperative assessment.

b Completeness calculated as percentage of total cases performed.

c Completeness figure given, but unable to identify method of calculation.

d Range of response rates between hospitals (national average not available).

e Planned starting collection at preoperative, 1, 6, and 10 years.

## Discussion

Our aim was to use international registry data to document trends in incidence rates of shoulder replacement and assess any recent changes in practice between countries.

We found that the incidence of shoulder replacement is rising in all registries, but remains lower than the incidence of hip and knee replacements [32]. However, the annual rate of growth in shoulder replacement (6–15%) outpaces the growth in primary hip (5.2%) and knee replacements (4.4%) [33]. The UK had the lowest incidence of shoulder replacement performed, but demonstrated the highest growth in the last 8 years. The rise in international incidence appears to be largely owing to the expanding indications, familiarity, and utilization of the RSTR, which is mirrored in other shoulder replacement epidemiological studies [34,35].

Over the last 5 years, the mean patient age at time of replacement has remained unchanged [1]. Although the gap is decreasing, replacement is still performed more frequently in women. The higher rate of procedures performed in women may be theorized to stem from the higher incidence of proximal humerus fragility fractures and arthritis in women [36]. The proportion of procedures performed for cuff tear arthropathy has increased. The absolute number and proportion of RTSRs performed has increased and largely replaced the use of HH. Germany and Norway did not show an increase in proportion of RTSR, however, as their proportion was similar to other registries; this suggests that they were early adopters, and potentially are now showing a steady state.

Compared with the previous study on this topic [1], RTSR is now universally performed for cuff tear arthropathy (pooled proportions have increased from 77% to 97%). Inter-registry variation exists in the treatment of OA, with a rise over the last 5 years in the use of RSTR to equal pooled proportions of TSR. This shift in practice does not seem to have been driven by high-quality RCTs. Some RCTs are now being commissioned to examine this question of using RSTR for primary OA with intact rotator cuff [37,38]. However, surgical practice seems to be changing already and hopefully equipoise will remain for a sufficient period for such trials to recruit and deliver.

Further change in practice can be observed from HH to RTSR for acute fracture over the last 5 years, with significantly higher proportional use of RTSR and decline in pooled proportions of HH from 68% to 29% [1]. This may have been driven by 2 RCTs, which demonstrated improved PROMs and satisfaction scores with RTSR [39,40]. However, while both RCTs were multi-centered, they had low sample sizes and a short mean follow-up of around 2.5 years. The use of TSR for trauma remains very low.

We observed a trend towards expanding the use of RTSR to other indications such as inflammatory arthropathy and AVN. While high-quality evidence is still lacking, it seems that an overall growing confidence in RSTR may be driving these changes.

Variation is seen in the use of stemless humeral components. Only 4 registries make a distinction between the use of stemmed or stemless humeral components. For HH, it appears that stemless humeral implants are becoming more popular at the expense of resurfacing humeral implants. Variation is observed in the use of stemless versus stemmed humeral implants for TSR. The reasons for these variations are unknown but possibilities include theoretically easier revision from stemless TSR to stemmed RSTR, or the use of stemmed modular “platform” systems that also argue for easier modular revision options.

The impact of COVID-19 on health services and the provision of shoulder replacement is worth commenting on, as considerable variation is observed between countries. While many countries seemed to, or needed to stop such surgery, it increased in Australia and NZ [41]. This appears to be due to an increase in shoulder replacements for fracture, a quicker return to provision of services, and a rebound effect in the latter half of the year. In contrast, the proportional drop-off in shoulder replacement surgery in the UK was 47.1%. This was mirrored in all other joints in the same registry that year, with the UK being the most affected country in Europe [42]. The pandemic therefore had a differing impact on elective surgical services in many countries, likely due to the differing COVID rates per country and differences in healthcare infrastructure and pandemic planning.

PROMs collection varied between countries regarding the instruments used, the timing of collection, the completeness achieved, and the way completeness was calculated. Completeness was predominantly below minimum recommended targets [43]. Harmonization of PROMs collection by international registries offers great potential for more meaningful shoulder replacement monitoring including benefit–risk assessment of established/new implants and surgical techniques, and benchmarking and outlier identification [44]. Harmonization—not only restricted to PROMs, but also applying to how revision rates are reported (e.g., cumulative risk of failure with 95% CI at yearly intervals) and which baseline characteristics are universally recorded—still has a long way to go, with more international collaboration between registries and governments required. Inclusion of complications not requiring revision, range of motion, and the combined use of shoulder function scores and disability scores could further improve shoulder replacement outcomes.

### Limitations

A major limitation was the inability to collect unbiased implant-specific data from 2020 onwards owing to COVID-19. Repeat assessment of variability when newer registry reports become available over the next few years would be useful, especially as a response to large multi-centered randomized controlled trials currently under way [37,45]. Future studies could also consider comparing more comprehensive data as registry reports continue to evolve, e.g., glenoid morphology and its impact on implant choice and variation based on demographic factors such preoperative activity, body mass index, or medical comorbidities.

Apart from completeness, the quality of data within each registry was not interrogated, meaning the validation processes and differences in definitions might lead to variation [46]. For example, there were inter-registry differences in categorization of indications, where indications such as AVN and chronic trauma were not listed in some registries and likely amalgamated into OA, altering its proportion. Conversely, patients with inflammatory arthropathies and coexisting osteoarthritis of the shoulder may have been inaccurately recorded.

Another limitation is that healthcare-related variations between countries were not assessed, such as healthcare systems, economic power, and availability of implants, all of which may affect the surgical choice and explain some of the variations observed [47].

Finally, as the majority of registries published only cumulative data, meta-analyses may have been skewed by the time of existence of each registry. Bigger shifts in practice might have been demonstrated if meta-analysis of the latest year was possible.

### Conclusion

We showed that the incidence of shoulder replacement surgery continues to rise internationally. There is less variation in the use of RSTR, which has become the dominant implant. Some variation still exists in other indications for shoulder replacement, although a definite growing trend is observed for the use of RTSR across these indications. Newer implants such as stemless humeral implants demonstrate variation between countries, while the use of shoulder-resurfacing implants is becoming obsolete. Patient-reported outcomes’ collection and completeness varies widely across all registries, limiting their use and questioning their ongoing collection without changes and improvements.

## Notes

(1) Conceptualization, (2) data curation, (3) formal analysis, (4) investigation, (5) methodology, (6) project administration and supervision, (7) validation, (8) writing original draft, and (9) review and editing. NR: 2, 3, 4, 5, 8, and 9; CC: 1, 3, 5, and 9; AS: 1, 4, 5, and 9; AL: 1, 2, 5, 6, and 9; JR: 1, 2, 4, 5, 6, 7, and 9

Handling co-editor: Taco Gosens

*Acta* thanks Frederik Okke Lambers Heerspink, Liza N van Steenbergen and other anonymous reviewers for help with peer review of this manuscript.
